# The Effects of Proton Pump Inhibitors in Acid Hypersecretion-Induced Vitamin B12 Deficiency: A Systematic Review (2022)

**DOI:** 10.7759/cureus.31672

**Published:** 2022-11-19

**Authors:** Kiran Maee Swarnakari, Meena Bai, Mohana Priya Manoharan, Rabab Raja, Aneeque Jamil, Denise Csendes, Sai Dheeraj Gutlapalli, Keerthana Prakash, Darshi M Desai, Aditya Desai, Safeera Khan

**Affiliations:** 1 Internal Medicine, California Institute of Behavioral Neurosciences & Psychology, Fairfield, USA; 2 Neuropsychiatry, California Institute of Behavioral Neurosciences & Psychology, Fairfield, USA

**Keywords:** proton pump inhibitors (ppi), gerd, vitamin b12 deficiency, elderly population, macrocytic anaemia, gastroesophageal reflux disorder (gerd), causes of vitamin b12 deficiency

## Abstract

Gastroesophageal reflux disease (GERD) is the most common disease, for which proton pump inhibitors (PPIs) are a widely used class of drugs. Due to their efficacy and relative safety profile, PPIs are used chronically by GERD patients. Although it is a safe drug, particular attention focuses on the long-term adverse effects of PPI. The association with vitamin deficiencies has received additional focus since chronic PPI treatment increases the incidence of vitamin B12 deficiency, especially in the elderly. However, numerous studies regarding the establishment of an association between PPI and vitamin B12 status revealed conflicting results.

In this systematic review, we systematically examined observational studies that focused on the impact of chronic PPI effects on vitamin B12 absorption and diagnostic biomarkers of vitamin B12 deficiency. Our review showed significant changes in diagnostic biomarkers of vitamin B12 status in long-term PPI users, including elevated homocysteine and methylmalonic acid (MMA) concentration levels defining cellular vitamin B12 deficiency. Although there is uncertainty regarding the exact mechanism, it supports the concept that long-term intake of PPI can have clinical implications for vitamins.

## Introduction and background

Gastroesophageal reflux disease (GERD) is the most commonly diagnosed gastrointestinal disorder in the adult population in the United States (US), with a prevalence of 20%. However, the true prevalence remains higher because many individuals have access to over-the-counter acid suppressants [1]. It is a chronic gastrointestinal disorder with a rising incidence in developed and developing countries, and it has been linked to the obesity epidemic and the aging population [1,2]. The American College of Gastroenterology recommends lifestyle changes as the primary treatment for GERD; however, moderate to severe GERD requires pharmacological treatment with medications that reduce gastric acid production. It includes antacids, histamine-2 receptor antagonists (H2RA), and proton pump inhibitors (PPIs) [1,3]. Due to their proven effectiveness and relative safety compared to histamine-2 receptor antagonists (H2RAs) [4, 5], PPIs are the most commonly prescribed medications to GERD patients worldwide [5, 6].

In 2012, more than 157 million PPIs were prescribed in the US, generating more than $13.5 billion in sales and thus remaining the third-largest-selling drug, resulting in a significant economic burden [7]. An analytical study of US noninstitutionalized adults by the Agency for Healthcare Quality and Research analyzed high-cost proton pump inhibitor (PPI) prescriptions and found $47.1 billion of excess expenditure over five years (2007-2011) [8], indicating a major economic burden on the healthcare system. Although PPI is an effective drug for GERD, it is associated with adverse effects [3]. Due to its long-term treatment by a larger population, its possible long-term adverse effects are receiving increasing attention [9]. Numerous studies have revealed minor adverse effects of PPI. One clinically significant area that is ignored is the effect of chronic acid suppression on the absorption of vitamins and nutrients [10]. This area has drawn significant attention because of the potential adverse effects of chronic PPI treatment leading to vitamin B12 deficiency. The area has been the subject of clinical trials, several systematic reviews, and meta-analyses [11]. Since then, the association between PPI intake and vitamin B12 deficiency has been supported by evidence.

The conclusion of several studies has revealed conflicting results. Some studies have shown that PPI use decreases vitamin B12 absorption [7,12]. Whether PPI can cause vitamin B12 deficiency still awaits clarification [11,13].

There is a lack of sufficient data to discuss PPI-induced vitamin B12 deficiency in chronic users. In addition, the definition of biochemical and clinical vitamin B12 deficiency, its diagnosis, and its diagnostic markers remain unclear. This study discusses the association between PPI and vitamin B12 deficiency. Thus, the context of this review describes vitamin B12 pathophysiology, its diagnostic markers, the proposed mechanisms for PPI-induced vitamin B12 deficiency, and its limitations.

## Review

Methods

In this systematic review, methods and findings were reported according to the Preferred Reporting Items for Systematic Reviews and Meta-Analysis (PRISMA) guidelines [14].

Search Strategy

We used an electronic research literature database and search engines such as PubMed, PubMed Central, Medical Literature Analysis and Retrieval System Online (MEDLINE), and Google Scholar to search for relevant articles using Medical Subject Headings (MeSH), in addition to a combination of keywords such as "proton pump inhibitors OR antacids OR acid suppression drugs AND Vitamin B12 OR cobalamin."

The following is a combined MeSH strategy conducted on PubMed, PubMed Central, and MEDLINE, as follows:( "Vitamin B 12 Deficiency/blood"[Majr] OR "Vitamin B 12 Deficiency/chemically induced"[Majr] OR "Vitamin B 12 Deficiency/diagnosis"[Majr] OR "Vitamin B 12 Deficiency/drug therapy"[Majr] OR "Vitamin B 12 Deficiency/etiology"[Majr] OR "Vitamin B12 Deficiency/organization and administration"[Majr] OR "Vitamin B 12 Deficiency/pathology"[Majr] OR "Vitamin B 12 Deficiency/physiopathology"[Majr] OR "Vitamin B 12 Deficiency/therapy"[Majr],) AND (( "Proton Pump Inhibitors/administration and dosage"[Majr] OR "Proton Pump Inhibitors/adverse effects"[Majr] OR "Proton Pump Inhibitors/etiology"[Majr] OR "Proton Pump Inhibitors/therapeutic use"[Majr] OR "Proton Pump Inhibitors/toxicity"[Majr] )

Inclusion and Exclusion Criteria

We considered observational studies, review articles published in English, focused on humans, and relevant to the research question. Case reports, letters, expert opinions, gray literature, unpublished literature, and studies focused on animals were excluded. A detailed description of inclusion and exclusion criteria is provided below (Table 1).

**Table 1:** Detailed inclusion and exclusion criteria

No	Inclusion criteria	Exclusion criteria
1	Papers published in the English language.	Papers not published in the English language.
2	Papers relevant to the research question.	Papers irrelevant to the research question.
3	Papers published on humans.	Papers published on animals; gray literature; unpublished literature.
4	Papers available in full text.	Papers that are not available in full text.
5	Papers focusing on the adult population (those over the age of 18).	Papers focusing on the pediatric population (18 years and under).
6	Observational studies, reviews, and meta-analyses are included.	Case reports, letters, expert opinions, and animal studies were excluded.

Literature Search and Study Design

Our search strategy of the PubMed, PubMed Central, and MEDLINE databases yielded 201 articles. After applying the appropriate filters (involving human studies, publications in the English language, abstracts, and book chapters) per our eligibility requirements, 161 articles were eliminated from the group, and duplicates were eliminated. The remaining publications (n = 40) were scrutinized by two researchers based on titles, abstracts, full texts, and specific inclusion and exclusion criteria.

**Figure 1:**
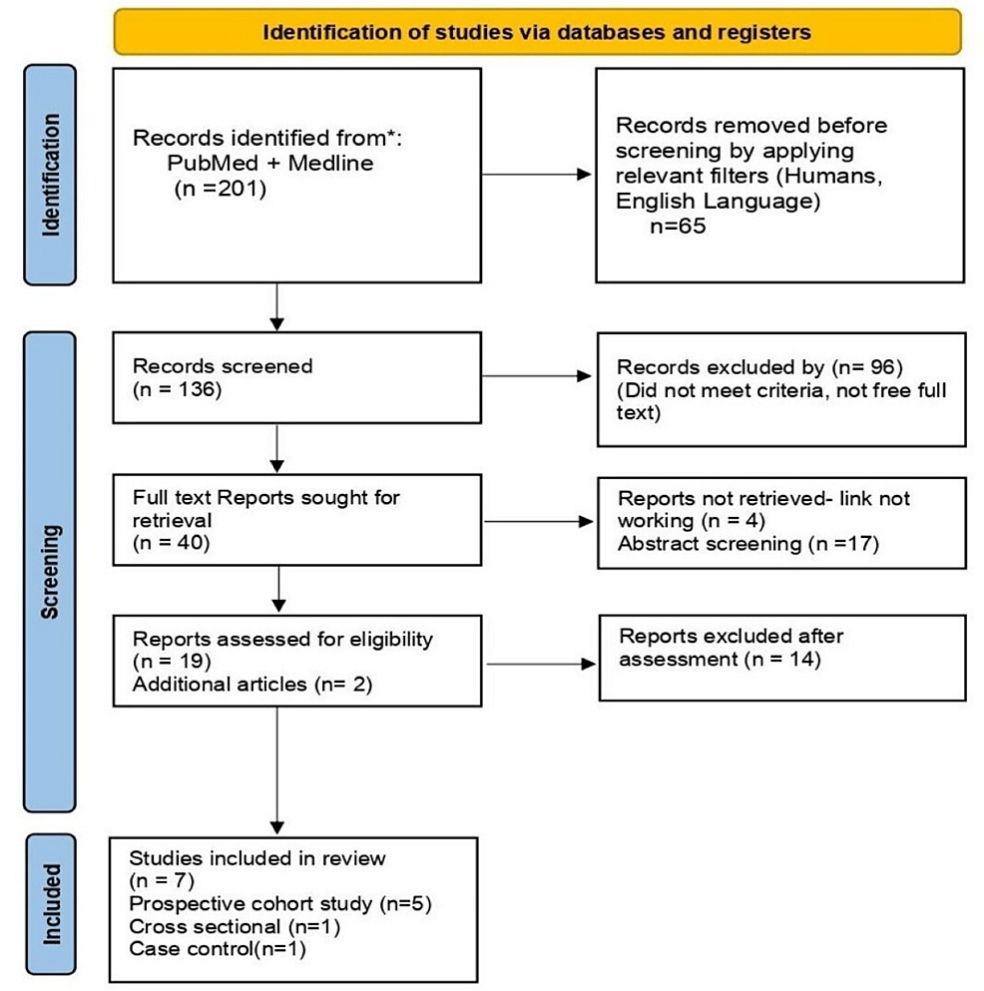
PRISMA flow diagram Preferred Reporting Items for Systematic Reviews and Meta-Analysis (PRISMA) Reference: [14]

After a thorough screening, we were left with 19 publications that addressed our research query. An additional two papers were found by looking up relevant terms in Google Scholar that were directly related to our topic. A comprehensive quality evaluation of 21 studies was conducted using established quality assessment techniques. After a quality evaluation, 14 studies were eliminated, leaving the remaining seven papers for this systematic review (Figure 1).

Quality Analysis of the Study

We critically evaluated the risk of bias using the Newcastle-Ottawa quality assessment scale for the observational studies (Table 2-4). We only included studies that qualified as medium or high quality on the Newcastle-Ottawa quality assessment scale for the observational studies.

The detailed scores and quality for each study are described (Table 2-4).

**Table 2:** Summary of the Newcastle- Ottawa Quality Assessment Scale for cohort studies

Selection	Porter KM, 2021 [15]	Qorraj- BH, 2018 [16]	Lewis JR, 2014 [17]	Hirschowitz, 2008 A [18]	Schenk, 2001 [19]
Representativeness of the exposed cohort	1	1	1	1	1
Selection of the non-exposed cohort	1	1	1	1	0
Ascertainment of exposure	0	1	1	1	1
Demonstration that the outcome of interest was not present at the start of the study	1	1	1	0	1
Comparability					
Study controls for the most important factor (age)	1	1	1	1	1
Study controls for any additional factor(s)	1	0	0	1	1
Outcome					
Assessment of outcome	1	1	1	1	1
Were follow-ups long enough for outcomes to occur?	1	1	1	1	1
Adequacy of follow-up of cohorts	0	1	0	1	0
Total	7/9	8/9	7/9	8/9	7/9
Quality	Moderate	High	Moderate	High	Moderate

**Table 3:** Summary of the Newcastle- Ottawa Quality Assessment Scale for case-control study

	Lam R. J, 2013 [7]
Selection	
Is the case definition adequate?	1
Representativeness of the cases	1
Selection of controls	1
Definition of controls	1
Comparability	
Study controls for (age)	1
Study controls for any additional factor	1
Exposure	
Ascertainment of exposure	1
The same method of ascertainment for cases and controls	1
Non-response rate	0
Total score	8/9
Quality of study	High

**Table 4:** Summary of Newcastle-Ottawa Quality Assessment Scale for cross-sectional study

	Den Elzen, 2008 [20]
Selection	
Representativeness of the sample	1
Selected group of users	1
Sample size	1
Diagnose	0
Comparability	
The study controls for the most important factor(age)	1
The study controls for any additional factor	1
Exposure	
Ascertainment of the method	1
Statistical test	1
Total score	7/8
Quality of study	High

Results

Baseline Characteristics of Included Studies

Of the seven included studies, five were prospective cohort studies, one was a cross-sectional study, and one was a case-control study. They included a total sample size of 48,108 participants, out of whom 27,653 were PPI users and 20,455 were controls (non-PPI users). These studies monitored vitamin B12 levels (total vitamin B12, methylmalonic acid (MMA), homocysteine, and holotranscobalamin (holoTC) levels) following the chronic use of proton pump inhibitors and evaluated the association between PPI use and vitamin B12 levels. Although these studies revealed conflicting results, there was a significant decrease in serum vitamin B12 levels in chronic PPI users in contrast to non-PPI users.

The following is a description of the baseline characteristics of the included studies (Table 5).

**Table 5:** Baseline characteristics of the included studies PPI: proton pump inhibitors; MMA: methylmalonic acid; holoTC: holotranscobalamin; Hcy: homocysteine; H2RAs: histamine receptor antagonists; ZES: Zollinger-Ellison syndrome; MCV: mean corpuscular volume; GERD: gastro-esophageal reflux disease; H. pylori: Helicobacter pylori

Author and year of publication	Interventions studied	Number of patients	Type of study	Primary outcome measured	Results
Porter KM, 2021 [15]	Proton pump inhibitors	Total sample size (3299), 1216 PPI users, and 2083 non-PPI users	Cohort study	Serum total vitamin B12, MMA, and holo-TC concentration levels	25% of PPI users have a high prevalence of vitamin B12 deficiency compared to 15% of controls (p<0.001). High PPI doses (≥30mg/d) associated with low holoTC, high MMA concentration (p<0.001)
Qorraj-B H, 2018 [16]	Proton pump inhibitors	Total sample size: 250; 200 long-term PPI users; 50 controls (non-PPI users).	Prospective cohort study	Baseline serum vitamin B12, iron, ferritin, and homocysteine levels after 12 months	For 12 months, there was no significant difference in vitamin B12, ferritin, and iron levels between PPI users and controls. After 12 months, there was a significant association and a 35.4% incidence of increased Hcy. However, 3.8% and 2.9% of the study group were diagnosed with hypoferremia and hypovitaminosis B12 at 12 months, respectively.
Lewis JR, 2014 [17]	Proton pump inhibitors	Total sample size: 94; 46 long-term PPI users; 48 controls (non-PPI users).	Prospective cohort study	Serum total vitamin B12 levels	Twenty three of 46 (50%) PPI users had low total serum Vitamin B12 levels in contrast to 10 of 48 (20.8%) non-PPI users, p = 0.003; PPI users had 18% lower total serum Vitamin B12 levels compared to controls. (p=0.030)
Lam J R, 2013 [7]	Proton pump inhibitors and H2 receptor antagonists	Total sample size (44,105), 25956 cases (vitamin B12 deficiency), and 18149 controls (no deficiency)	Case-control study	Vitamin B12 deficiency	≥2 yrs. of PPIs (OR, 1.65) and H2 RAs (OR, 1.25) associated with vitamin B12 deficiency.
Hirschowitz, 2008 [18] A	Proton pump inhibitors	61 acid hypersecretors (46 ZES, 15 others) are taking PPI.	Longitudinal study	Yearly Vitamin B12 levels	Six of 61 (10%) had low vitamin B12 levels without signs of vitamin B12 deficiency. 13 of 41 people (31% of the total) had normal vitamin B12 levels but higher MMA and hydroxycitric acid levels with normal folate levels. Prolonged acid suppression does not explain vitamin B12 deficiency.
Den Elzen, 2008 [20]	Proton pump inhibitors	Total sample size: 250; 125 long-term (> 3 years) PPI users; 125 partners (non-PPI users).	Cross-sectional study	Serum vitamin B12, homocysteine levels, and MCV	There was no difference in vitamin B12 levels between PPI users and nonusers. Furthermore, there was no difference in homocysteine or MCV levels. However, 2.3% of users and 2% of nonusers had low vitamin B12 levels.
Schenk BE, 2001 [19]	Proton pump inhibitors	49 PPI users (H. pylori-positive GERD)	Prospective cohort study	Serum cobalamin levels, atrophic gastritis	Fifteen of 49 (33%) PPI users developed atrophic gastritis, of whom nine had moderate-to-severe atrophy. There was a significant decrease in serum cobalamin levels in patients with atrophic gastritis (more pronounced in nine PPI users). However, no difference was reported between atrophic gastritis and non-gastritis concerning age, dose, or duration.

Discussion

Role of Vitamin B12

Vitamin B12 is a water-soluble vitamin that serves as a cofactor for enzymes. Vitamin B12 plays a significant role in DNA synthesis and amino acid metabolism as a cofactor. This role is essential for erythropoiesis and myelination of the nervous system.

Vitamin B12 is mainly obtained through the dietary consumption of animal products, dairy products, and vitamin supplements [9,10]. Dietary vitamin B12 is absorbed by two processes. The first process occurs through the intestinal mechanism, and the second is through passive diffusion. Most of the dietary vitamin B12 is absorbed in the intestines using an intrinsic factor. This intestinal mechanism allows for the absorption of 1-2 micrograms of vitamin B12 every few hours. Digestive proteases in the stomach and small intestine are required to release protein-bound vitamin B12 from the proteins [4,9]. In the stomach, pepsin releases B12 from food proteins. Then it is bound by R-protein, which is produced in the salivary glands (also known as haptocorrin and transcobalamin-1). This guards against vitamin deterioration in the stomach's acidic environment [11]. In the duodenum, R-protein is degraded, and vitamin B12 binds to intrinsic factors. This combined complex is ingested by enterocytes of the distal ileum and then binds to transcobalamin (a blood carrier protein) to be delivered peripherally [9].

Proton Pump Inhibitors-Induced Vitamin B12 Deficiency

The association between PPI and vitamin B12 has been determined in numerous studies. Many possible theories were proposed to explain their interaction, leading to vitamin B12 deficiency. The most accepted theory for vitamin B12 absorption is that intact gastric corpus mucosa that harbors oxyntic glands, including parietal cells, is necessary [9].

PPIs work by blocking gastric H+K+-ATPase, which is responsible for pumping H+ ions from within gastric parietal cells into the gastric lumen, where they react with Cl ions to form hydrochloric acid [10]. As described above, pepsinogen is converted to pepsin by gastric acid to release vitamin B-12 from food proteins. A lack of gastric acid due to PPI or histamine H2 receptor antagonist (H2RA) use (or pathophysiologic conditions that affect gastric acid production, such as atrophic gastritis) will reduce the digestive capacity to release vitamin B-12 from foods and thus reduce the amount of vitamin B-12 that is absorbed in the body [6,10,11]. 

Proton Pump Inhibitors and Vitamin B12 Interaction

The side effects of chronic PPI intake often go neglected due to distinct differences in the diagnosis of vitamin deficiencies. In this systematic review, we analyzed the study outcomes, the pathophysiology of vitamin B12 deficiency, and potential limitations.

The most recent 2021 cohort study examined 3299 community-dwelling older adults (1216 PPI users, 2083 non-PPI users) and the protective role of fortified foods on vitamin B12 levels [15]. The study concluded that participants with higher PPI doses (≥30 mg/d) for more than six months were significantly associated with a higher prevalence of vitamin B12 deficiency (21% compared with 15% in controls; p = 0.001), lower holotranscobalamin (holoTC), and high MMA levels. There was a significantly higher prevalence of vitamin B12 deficiency in unfortified food participants compared to vitamin B12-fortified food intake (crystalline form). Furthermore, the author concluded that fortified food consumption benefited older individuals with chronic, higher doses of PPI [15]. 

The findings of a large population-based study in 2013 identified >25,956 vitamin B-12-deficient cases and 184,199 controls using an electronic pharmacy database to determine PPI exposure [7]. The risk of a subsequent diagnosis of vitamin B-12 deficiency was shown to be greater at higher PPI treatment levels (i.e., 95% risk of deficiency with >1.5 pills/d compared with a 63% risk with <0.75 pills/d). However, the PPI dose per se was not reported. The limitations of this study are due to its design; an observational study can only provide association, unlike causation.

The above research studies emphasize the significance of taking PPI dosage into account when considering its association with vitamin B-12 status [15,7]. The impact of PPI intake over a longer period (than the six months that are being considered here) is also necessary to demonstrate how this drug affects long-term vitamin B12 status and whether routinely checking vitamin B12 status in PPI users should be advised.

A 2018 cohort study examined 250 adult patients (200 PPI users and 50 non-PPI users) who had been taking pantoprazole (40 mg/day), omeprazole (20 mg/day), esomeprazole (20 mg/day), and lansoprazole (30 mg/day) for at least a year [16]. It found that PPI use for one year was significantly associated with a decrease in vitamin B12 levels and that PPI users had a 39.5 percent higher incidence of hyperhomocysteinemia than non-PPI users. The study's limitations include the inability to determine blood MMA levels and evaluate vitamin B12 food consumption.

The 2014 cohort research, which included 94 postmenopausal women living in the community, provides evidence supporting the idea that extended PPI usage reduces vitamin B12 absorption [17]. A study involving 46 PPI users and 48 non-PPI users showed low total blood vitamin B12 levels and a 50% greater prevalence of vitamin B12 insufficiency among PPI users, suggesting a potential mechanism for PPI-impaired malabsorption as a result. The research's postmenopausal study sample makes it impossible to extrapolate the results to the general population, which is one of the study's limitations.

In a 2008 longitudinal study involving 61 participants with hypersecretion (basal acid production > 15 mmol/h), it was shown that long-term PPI treatment raised the risk of vitamin B12 insufficiency by 29% [18]. In particular, patients with acid hypersecretors on PPI treatment had a greater than expected frequency of B12 insufficiency. As a result of high levels of homocysteine and MMA and their reaction to B12 supplementation, 13 of 41 patients (31%), whose serum vitamin B12 levels were also determined to be probably deficient.

Similar cross-sectional research was done in 2008 with 300 participants (125 long-term PPI users (>3 years) and 125 non-PPI users) [20]. The study, according to the author, was neither associated with vitamin B12 deficiency nor with clinical indicators of it, such as elevated homocysteine levels (mean difference = 0.4 (95 percent CI: 1 to 0.5), P = 0.42) and elevated mean corpuscular volume (MCV) (adjusted mean difference = 0.1 (95 percent CI: 0.9 to 1), P = 0.88). Even though both research studies produced contradictory findings, the contradictory results of the two earlier studies on the relationship between PPIs and vitamin B-12 insufficiency may be attributable to differences in the research designs, study populations, and the use of biomarkers to assess vitamin B-12 status and define deficiency [18,20].

In a 2001 prospective cohort study of 49 PPI users, 33% were found to have low blood cobalamin levels, and 60% of these people developed moderate-to-severe atrophic gastritis with persistent H. pylori infection [19]. 15 out of 49 patients who developed atrophic gastritis had a substantial decline in serum vitamin B12 levels (Vitamin B12: 61 vs. +33, P< 0.01); the impact was most prominent in the nine patients who experienced moderate to severe atrophy. In contrast to omeprazole maintenance therapy, a drop in serum cobalamin levels was seen in individuals with atrophic gastritis [19].

Diagnosis of Vitamin B12 Deficiency

The laboratory diagnosis of vitamin B12 deficiency has always been difficult. Diagnostic tests depend on two factors, i.e., to measure levels of circulating vitamin B12 and to measure levels of serum markers that accumulate due to its deficiency. Circulating vitamin levels are determined by serum vitamin B12 and holoTC, whereas MMA and homocysteine levels are increased when vitamin B12 deficiency exists [10]. A disadvantage of laboratory diagnosis is the lack of a gold standard test to assess vitamin B12 levels in order to diagnose deficiency.

In line with some earlier research results, no significant association was found using serum total vitamin B12 as a biomarker. However, results show a significant association when using serum homocysteine levels as biomarkers for deficiency [18,16,20,21]. Therefore, we can conclude that diagnosing B12 deficiency based solely on serum B12 levels leaves many cases undiagnosed. The more specific of the two markers of B12 deficiency, homocysteine (Hcy), and MMA, is elevated MMA. Elevated MMA suggests a vitamin B12 deficiency [22]. According to a recent study, holoTC is a promising first-line test for determining vitamin B12 deficiency. Before establishing a standard diagnostic approach with pertinent cut-off values, it is necessary to do more research to determine whether to employ holoTC as the first-line test or combine it with a metabolic marker [23].

Limitations 

An important limitation of our systematic review was the lack of a randomized clinical trial (RCT); no eligible RCTs satisfied the quality check and eligibility criteria. This study considered seven observational studies after a quality check. It raises further concern about conducting an RCT to strengthen the evidence. Observational studies include selection bias, which is another limitation. In addition, this study reviewed articles focusing on the geriatric population, raising concerns about external validity, which does not apply to the general population.

## Conclusions

Long-term and high-dose PPI use may result in a vitamin B12 deficiency. Clinical symptoms of vitamin B12 deficiency range from modest anemia to severe neurodegenerative deficits. A laboratory marker of deficiency is determined by elevated homocysteine levels, which are significantly associated with vascular and neurodegenerative deficits. Furthermore, it has the potential to increase the risk of cardiovascular disease. The differences in diagnostic markers and their values are probably a factor behind the remarkable variation in prevalence. As a result, the diagnostic markers of vitamin B12 deficiency require special attention to establish deficiency before clinical manifestations.

Hence, this study will aid physicians to be cautious regarding PPI-induced vitamin deficiencies, judiciously use PPI, and discontinue it whenever it is not required. When prescribing long-term PPI, a prompt diagnosis of vitamin deficiency is critical, especially in elderly patients who are more susceptible. However, there is a need for further research to investigate PPI-induced vitamin B12 deficiency, particularly randomized clinical trials, to monitor the adverse effects of long-term usage of PPI. Furthermore, more prospective research into the clinical consequences of proton pump inhibitor-induced vitamin B12 deficiency may be beneficial. 
